# Invasive Group B Streptococcal Infections in Infants, France

**DOI:** 10.3201/eid1410.080185

**Published:** 2008-10

**Authors:** Claire Poyart, Hélène Réglier-Poupet, Asmaa Tazi, Annick Billoët, Nicolas Dmytruk, Philippe Bidet, Edouard Bingen, Josette Raymond, Patrick Trieu-Cuot

**Affiliations:** Institut National de la Santé et de la Recherche Médicale U567 Unité Mixte de Recherche Centre National de la Recherche Scientifique 810, Paris, France (C. Poyart, H. Réglier-Poupet, A. Tazi); Centre National de Référence des Streptocoque, Paris (C. Poyart, H. Réglier-Poupet, A. Tazi, N. Dmytruk, P. Bidet, E. Bingen, J. Raymond, P. Trieu-Cuot); Groupe Hospitalier Cochin-Saint Vincent de Paul, Paris, (C. Poyart, H. Réglier-Poupet, A. Tazi, A. Billoët, J. Raymond); Institut Pasteur, Paris (C. Poyart, P. Trieu-Cuot)

**Keywords:** Streptococcus agalactiae, GBS, invasive infections, meningitis, ST-17, dispatch

## Abstract

Clinical features and molecular characterization of 109 group B streptococci causing neonatal invasive infections were determined over an 18-month period in France. Sixty-four percent of the strains were from late-onset infections, and 75% were capsular type III. The hypervirulent clone ST-17 was recovered in 80% of meningitis cases.

Group B *Streptococcus* (GBS) is the leading cause of infectious illness among newborns. Invasive infections in neonates can result in pneumonia, sepsis, or meningitis. Early-onset disease (EOD) occurs within the first week. Late-onset disease (LOD) occurs after the first week and accounts for most meningitis cases and deaths (*1*). Because recommendations for intrapartum antibioprophylaxis (IAP) for mothers in labor at risk for GBS infection have been widely implemented in many countries, the incidence of EOD has declined to <1/1,000 births, but the incidence of LOD has remained unchanged (*2*). To date, 10 capsular serotypes have been described (Ia, Ib, and II–IX). Among these, serotype III is of particular importance because it is responsible for a substantial proportion of EOD and most cases of LOD (*3*–*8*). Different studies have suggested that most neonatal invasive diseases and almost all cases of meningitis are caused by a limited number of strains belonging to a homogeneous serotype III clone. This clone is defined by multilocus sequence typing (MLST) analyses as ST-17, the so-called highly virulent clone (*4*–*8*). However, data available in Europe are limited regarding the distribution of GBS genotypes among invasive isolates recovered from neonates.

We describe clinical characteristics, capsular type, and MLST allelic and antimicrobial drug-susceptibility profiles of 109 nonredundant GBS isolates that caused neonatal invasive infections. These isolates were collected during an active surveillance performed in France from May 2006 through December 2007.

## The Study

Clinical data on 109 infants up to 4 months of age were analyzed. Sepsis was defined as GBS bacteremia in the presence of consistent clinical signs and symptoms. Meningitis was diagnosed if GBS was recovered from cerebrospinal fluid. GBS isolates were identified by using a commercial Lancefield group–specific latex agglutination test. Capsular typing was performed by a multiplex PCR as described (*9*), and the hypervirulent ST-17 clone was detected by real-time PCR, as reported (*6*). Susceptibility testing, antibiograms, and MICs were performed according to Clinical and Laboratory Standards Institute recommendations (www.clsi.org). Antimicrobial drug–resistance genes were detected by using the multiplex PCR as described (*10*). Statistical analysis was performed according to the Fisher exact and χ^2^ tests. A p value of <0.05 was used as the threshold for statistical significance.

We studied 109 GBS strains responsible for neonatal invasive infections; 36% (n = 39) and 64% (n = 70) were responsible for EOD and LOD, respectively (Table). Eighty percent of EOD cases occurred during the first 24 hours after birth, with a male:female ratio of 0.9; 72% were associated with sepsis, and 28% with meningitis. Maternal cultures obtained in the last 6 weeks before delivery were available for 64% of the cases. Positive GBS cultures were detected in only 11% of the cases. One death associated with meningitis occurred in an infant with EOD. A capsular serotype was assigned to all isolates with a distribution as follows: types III (61.5%) and Ia (28.2%) were predominant compared to types Ib (5.1%), II (2.5%), and V (2.5%) (Table). Capsular types IV, and VI–IX were not found. EOD meningitis GBS strains were of type III in 81.8% of isolates, and all these strains were ST-17 positive.

**Table:** Characterization of the 109 GBS strains isolated from neonatal invasive infections, France, 2006–2007*

Origin of strains (no. isolates)	CPS (no. isolates)					
	Ia (16)	Ib (7)	II (1)	III (82)	V (3)	ST-17 (75)	
EOD <7 d (39)							
Sepsis (28)	9	2	1	15	1	13	
Meningitis (11)	2	0	0	9	0	9	
LOD >7 d (70)							
Sepsis (19)	2	1	0	15	1	13	
Meningitis (46)	3	3	0	40	0	37	
Other† (5)	0	1	0	3	1	3	

*GBS, group B *Streptococcus*; CPS, capsular serotype; EOD, early-onset disease; LOD, late-onset disease. †Sepsis was associated with 2 cases of parotitis, 1 case of osteomyelitis, 1 of spondylodiscitis, and 1 of orchitis.

LOD had a male:female ratio of 1.15, and 82.6% of cases occurred during the first 8 weeks of life, with a peak (63%) at 4–8 weeks (data not shown). Sepsis occurred in 27.1% of LOD cases and meningitis in 65.7%. In 5 cases of LOD, less frequent manifestations were observed: sepsis was associated with parotitis (2 cases), osteomyelitis (1), spondylodiscitis (1), and orchitis (1). Three cases (4.5%) of recurrent invasive infections were reported. For 2 of these 3 cases, the first episode was early meningitis with a relapse of meningitis 2–3 weeks later, despite correct antimicrobial drug treatment. The third case was a late-onset sepsis that relapsed as a sepsis after the infant had received 3 weeks of amoxicillin. None of these infants was fed breast milk, which ruled out the possibility of contamination by this route. The death rate for LOD was 14.5%; 90% of deaths were associated with meningitis. Capsular type distribution of GBS LOD isolates was as follows: type III was largely predominant (83%) compared with types Ia (7.4%), Ib (4.5%), and V (1.5%) (Table). Among strains responsible for meningitis, 87% were of type III and almost all (92.5%) belonged to the hypervirulent ST-17 clone.

All 109 GBS strains tested were susceptible to penicillin (MIC_90_ 0.016 mg/L), amoxicillin (MIC_90_ 0.016 mg/L), cefotaxim (MIC_90_ 0.016 μg/mL), imipenem (MIC_90_ 0.032 μg/mL), rifampin (MIC_90_ 0.032 μg/mL), vancomycin (MIC_90_ 0.75 μg/mL), and displayed low-level resistance to gentamicin (MIC_90_ 8 μg/mL). Also, 95.5% were resistant to tetracycline because of the presence of *tet*(M) associated with *tet*(O) or *tet*(L) in 3 and 1 strains, respectively. Resistance to erythromycin was detected in 13.8% of the isolates and was not correlated with the capsular type or the onset of disease. Erythromycin resistance was caused by the presence of *mef*(A) (46.6%), *erm*(A) (26.6%), or *erm*(B) (20%).

## Conclusions

In France, screening of pregnant women for GBS colonization and IAP for women detected positive was implemented in 2001 but, despite these recommendations, EOD continues to occur (*11*). In this report, 36% of cases were EOD. For 71% of EOD cases, maternal vaginal screening before delivery had not been conducted or was negative for GBS, thus likely explaining the persistence of EOD, as already suggested by others (*12*,*13*). In our study, LOD represents the majority of cases (64%), which is consistent with findings in countries where a screening approach, together with IAP, was adopted (*2*,*11*,*13*).

Clinical symptoms were significantly associated with the time of infection onset: EOD was mostly associated with sepsis (72%), whereas LOD was more frequently responsible for meningitis (65.7%) (p<0.01). Deaths, all associated with meningitis, were higher in LOD (14.5%) than in EOD cases (2.5%).

The predominance of capsular type III among infants with meningitis is well-known (*3*,*5*–*8*,*14*,*15*). In our study, type III accounted for 83% of LOD and was significantly associated with meningitis (85.9%; p<0.01) in both EOD and LOD. Moreover, the hypervirulent clone ST-17 was significantly predominant among LOD cases (75%; p<0.03) and accounted for 93% of GBS type III strains responsible for meningitis. This overrepresentation of ST-17 among invasive neonatal strains is now well recognized worldwide and highlights the fact that this clone is well adapted to neonate pathogenesis and may possesses specific virulence traits that enhance its invasiveness in this population (*5*–*8*,*14*,*15*). Early detection of this clone among colonizing strains in pregnant women or in neonates at delivery may therefore constitute the basis for developing new prevention strategies. An attractive alternative to IAP is vaccinating young women to subsequently protect neonates against GBS infections. Conjugate vaccines composed of capsular polysaccharides and tetanus toxoid have already been evaluated (*1*). Recent studies have suggested that protein antigens induce protective immunity in animal models, and surface proteins common to many strains would have a potential role in vaccine development (*1*). For this reason immunogenic antigens specific to the ST-17 clone should be considered in designs of future vaccine.
